# 

**Published:** 1888-12

**Authors:** 


					﻿Died.—Dr. Sylvester L- Bedall, at Eagle Pass, November 18,
1888, of lead poisoning and obstruction of bowels.
Married—In Bristol, Tenn., December —, Dr. F. A. Max-
well, of Del Valle, Texas, to Miss Ella King, of Tennessee.
Died.—In Brownsville, Texas, November—ult., Dr. Etienne
Melou, a prominent and much respected practitioner of that city.
See memoriam resolutions passed by the B. and M. Med. Society.
Married.—In Meridian, Texas, November 28, (ult.,) Dr.
Nelson A. Olive to Miss Eavinia, daughter of Mr. and Mrs. W.
T. Campbell, all of that city. Dr. Olive is a member of the
Texas State Medical Association.
We acknowledge the courtesy of an invitation to the marriage;
and extend to the handsome young doctor our congratulations
and sincere well wishes.
Death of Dr. White, of Cuero.—As we go to press we hear
the sad tidings of the death of Dr. A. C. White, of Cuero.' He
died December 5, in the 53rd year of his age. Dr. White was a
native of Virginia, a graduate ot the New Orleans Medical
College, and a Confederate Surgeon in Sibley’s brigade during
the war. He was well known as one of the leading physicians
of South Texas, and was a valued member of the Texas State
Medical Association. He leaves a wife and five children.
Died—In Louisville, Ky., December 9, (instant,) Dr. W. R.
Spaulding, late of Austin, Texas. Dr. Spaulding studied medi-
cine in this city with Dr. J. W. McLaughlin, and graduated at
Louisville, Ky., with first honors last year, winning many prizes.
He also won the position of resident physician in the hospital
by competitive examination, the position he was filling at the
time of his death. [The death of this promising young physician
is much to be deplored, as he would have shone in his profession
and made his mark, had he lived. The remains were brought
to Austin for interment.
“El Paso, as a Health Resort,” is the caption of a very able
paper by Doctor Wm. M. Yandell of that city, addresssd to a
committee of enterprising citizens, and published, we presume,
for general distribution.
The doctor, with great care and patience, studied the tables of
the signal service, and upon the records, based a scientific argu-
ment in favor of El Paso, that must carry conviction to every
reader.
We congratulate the people of that favored spot upon their
natural advantages, and upon having a health officer who can so
clearly demonstrate the fact to the outside world.
[Book Notices and Cullings from Exchanges crowded out.—
Ed.]
				

